# Single Molecule Study of the Polymerization of RecA on dsDNA: The Dynamics of Individual Domains

**DOI:** 10.3389/fmolb.2021.609076

**Published:** 2021-03-22

**Authors:** Nitzan Maman, Pramod Kumar, Amarjeet Yadav, Mario Feingold

**Affiliations:** ^1^ Department of Physics, Ben Gurion University of the Negev, Beer Sheva, Israel; ^2^ The Ilse Katz Center for Nanotechnology, Ben Gurion University of the Negev, Beer Sheva, Israel; ^3^ Department of Applied Physics, Babasaheb Bhimrao Ambedkar University, Lucknow, India

**Keywords:** single molecule, recA, optical tweezers, protein-DNA interaction, nucleation and growth

## Abstract

In the *Escherichia coli*, RecA plays a central role in the recombination and repair of the DNA. For homologous recombination, RecA binds to ssDNA forming a nucleoprotein filament. The RecA-ssDNA filament searches for a homologous sequence on a dsDNA and, subsequently, RecA mediates strand exchange between the ssDNA and the dsDNA. *In vitro*, RecA binds to both ssDNA and dsDNA. Despite a wide range of studies of the polymerization of RecA on dsDNA, both at the single molecule level and by means of biochemical methods, important aspects of this process are still awaiting a better understanding. Specifically, a detailed, quantitative description of the nucleation and growth dynamics of the RecA-dsDNA filaments is still lacking. Here, we use Optical Tweezers together with a single molecule analysis approach to measure the dynamics of the individual RecA domains on dsDNA and the corresponding growth rates for each of their fronts. We focus on the regime where the nucleation and growth rate constants, *k*_*n*_ and *k*_*g*_, are comparable, leading to a coverage of the dsDNA molecule that consists of a small number of RecA domains. For the case of essentially irreversible binding (using ATPγS instead of ATP), we find that domain growth is highly asymmetric with a ratio of about 10:1 between the fast and slow fronts growth rates.

## Introduction

The primary function of the RecA protein is to exchange strands as part of the homologous recombination process in *Escherichia Coli* (Radding, 1988; Cox, 1999; Kowalczykowski, 2000; Cox, 2007b; Prentiss et al., 2015; Bell and Kowalczykowski, 2016). In addition, RecA plays an important role in DNA repair. One of the first steps in homologous recombination is the assembly of RecA-ssDNA filaments, whereby each RecA monomer attaches to three DNA base pairs. At the next stage, a RecA-ssDNA filament searches for a homologous sequence on the target dsDNA. Once such sequence is located, RecA induced strand exchange occurs, whereby the ssDNA replaces the homologous strand of the dsDNA. *In vitro*, RecA can polymerize on either ssDNA or dsDNA. The RecA-DNA filament is significantly more rigid than the bare DNA. Its formation requires ATP and a divalent cation e.g. Mg^2+^. Polymerization of RecA on DNA takes place via the growth of domains. The first few protein monomers that bind at adjacent sites in a protein free region of the DNA form a nucleus which subsequently grows by adding monomers to each of the two sides of the new domain. The growth of domains is asymmetric, being faster in the 5′ to 3′ direction of the DNA. While for ssDNA nucleation and growth are relatively fast, when the protein binds to dsDNA the nucleation step is much slower (Pugh and Cox, 1988). In the case of dsDNA, the presence of single strand regions, e.g., a nick, accelerates the nucleation step. Nucleation on dsDNA is also accelerated by stretching the molecule or reducing the pH. In contrast, the secondary structure of dsDNA restricts the attachment of the RecA preventing full coverage of the DNA. Structurally, the effect of RecA binding is to extend the length of each group of three base pairs (bp) by a factor of about 1.5 and unwind the dsDNA from a 35° twist down to one of only 20° (Stasiak and Egelman, 1994). Proteins that are homologous to RecA are found in all species, e.g., the human Rad51 protein (Baumann and West, 1998).

The RecA protein has been widely studied using biochemical methods and many of its properties are known. More recently, single molecule methods were introduced that allow measuring directly properties of a particular molecule which are lost at the macroscopic scale (Leger et al., 1998; Hegner et al., 1999; Shivashankar et al., 1999; Sattin and Goh, 2004; van der Heijden et al., 2005; Galletto et al., 2006; Joo et al., 2006; Mine et al., 2007; van Loenhout et al., 2009; Danilowicz et al., 2012; De Vlaminck et al., 2012; Forget and Kowalczykowski, 2012; Fu et al., 2013a; Fu et al., 2013b; Lee et al., 2013; Kim et al., 2014; Kim et al., 2015; Lee et al., 2015; Qi et al., 2015; Lee et al., 2016; Danilowicz et al., 2017; Lee et al., 2017). For example, magnetic and optical traps have been used to study the effect of external force and torque on the polymerization of proteins on DNA and for testing models of homology search strategies (Leger et al., 1998; Hegner et al., 1999; Shivashankar et al., 1999; van der Heijden et al., 2005; Mine et al., 2007; van Loenhout et al., 2009; Danilowicz et al., 2012; De Vlaminck et al., 2012; Forget and Kowalczykowski, 2012; Fu et al., 2013a; Fu et al., 2013b; Lee et al., 2013). Moreover, quick dynamic changes during the RecA-DNA filament formation were measured using fluorescence resonance energy transfer (FRET) (Joo et al., 2006). FRET also allows monitoring the kinetics of the RecA mediated strand exchange process by selectively labeling each of the two strands with different fluorophores, a donor and an acceptor (Kim et al., 2014; Kim et al., 2015; Danilowicz et al., 2017).

In the assembly of the RecA-dsDNA filament, the nucleation and growth rates strongly depend on the environmental chemical and physical conditions. Measuring the rate of ATP hydrolysis, it was shown that the rate of RecA binding is larger for a dsDNA tailed with a 5′ ssDNA than one tailed with a 3′ ssDNA (Register and Griffith, 1985; Lindsley and Cox, 1990). This indicates that the growth of RecA domains on DNA is asymmetric with a strong preference to the 5′ to 3′ direction. In the light of these results, it was suggested that RecA-dsDNA filaments that form in the presence of the essentially non-hydrolysable ATPγS (Weinstock et al., 1981) should behave differently, namely, their assembly would proceed in a symmetric manner (Cox, 2007a). Apparently, this expectation relied on the assumption that the RecA-ATP complex has a different configuration when attached to DNA and this configuration change is due to the ATP hydrolysis.

Biochemical methods, as those described in the previous paragraph, cannot provide detailed information regarding the dynamics of the individual RecA domains on a particular dsDNA molecule. Such domain dynamics can be extracted using a single DNA approach that exploits the fact that RecA binding leads to the local extension of the dsDNA length. In particular, one can extract the coverage dynamics of RecA on dsDNA from the time dependent growth of the dsDNA contour length during RecA polymerization. This approach was used to determine the rate constants of nucleation, $k_{n}$, and growth, $k_{g}$, of RecA on individual dsDNA molecules (Leger et al., 1998; Hegner et al., 1999; Shivashankar et al., 1999). Some of these experiments were performed in the range of high nucleation rate where it is difficult to separate nucleation from growth such that only the combined rate constant, $k_{n}k_{g}$, could be measured (Shivashankar et al., 1999). Nevertheless, it was subsequently shown that one can use a nucleation and growth molecular model to simulate the experimentally measured RecA polymerization dynamics and extract each of the individual rate constants, $k_{n}$ and $k_{g}$, separately (Turner, 2000). A similar approach was also used to study the assembly of the Rad51 recombinase on DNA (Mine et al., 2007; Pierobon et al., 2010).

Single molecule methods were also used to study the effect of force applied to the DNA on the rate of RecA polymerization and the efficiency of the homology search (Leger et al., 1998; van Loenhout et al., 2009; Danilowicz et al., 2012; De Vlaminck et al., 2012; Fu et al., 2013a; Fu et al., 2013b). In the experiments of Leger et al. (Leger et al., 1998), the dsDNA molecule was stretched with constant force (10–100 pN) and the elongation rate due to RecA binding was monitored. It was shown that the RecA-dsDNA complex formed more rapidly in the presence of applied force that reduced the energy barrier for protein binding. For example, stretching the dsDNA with a force of 75 pN lead to a reaction rate that was 20 times faster than that observed when a 15 pN force was applied. Fu et al. (Fu et al., 2013b) used magnetic tweezers to study the competition between RecA polymerization and de-polymerization on dsDNA for different temperatures, pH values and salt concentrations. They showed that these parameters distinguish between a regime where RecA polymerization is stable and another where it is transient reverting to naked dsDNA at the end of the process. Magnetic tweezers were also used to control the torque applied to the dsDNA and probe its effect on the assembly of RecA or Rad51 onto dsDNA (van der Heijden et al., 2005; Lee et al., 2013). van der Heijden et al. (van der Heijden et al., 2005) found that the polymerization process stalls at high torsions of the dsDNA molecule leading to dsDNA molecules that are only partially covered with RecA.

The different stages of homologous recombination were also studied at the single molecule level using fluorescence microscopy (Galletto et al., 2006; Forget and Kowalczykowski, 2012; Lee et al., 2015; Qi et al., 2015; Lee et al., 2016; Lee et al., 2017). For example, Galletto et al. (Galletto et al., 2006) analyzed the assembly of individual RecA-dsDNA filaments. They used fluorescently labeled RecA to image the segments of the dsDNA that were coated with RecA at intermediate stages of the polymerization reaction. In agreement with previous results from biochemical studies, they showed that RecA polymerization is controlled by the slow nucleation step. When ATP was used in the reaction, the corresponding critical nucleus is about four–five protein monomers. In contrast, the growth of the RecA domains is a relatively fast process for both of the cofactors that were studied, namely, ATP and ATPγS. The asymmetry of RecA domain growth was also analyzed in the presence of either ATP or ATPγS. Although for both cofactors the data indicated a certain extent of asymmetric growth, it was not sufficiently accurate to allow for the quantitative determination of the fast and slow growth rates.

In this paper, we present a new approach to measuring the growth rates of individual RecA domains on dsDNA that allows obtaining the growth rate for each of the two fronts of individual domains. This enables us to establish the extent of asymmetry between the fast and the slow fronts of each domain. To this end, we restrict our study to the case of essentially irreversible binding (ATPγS) (Lindsley and Cox, 1989). Biochemical studies indicate that ATP hydrolysis is only required in the final phase of RecA dissociating from the DNA and, accordingly, using ATPγS instead of ATP has become a standard approach in the study of the reaction between RecA and DNA (Weinstock et al., 1981). Similarly, in our study, ATPγS was used in order to separate the assembly phase of the RecA on dsDNA from that of the disassembly. Moreover, we choose the experimental parameters such that the nucleation and growth rates are comparable, $k_{g}/k_{n}\approx 1$. In this regime, we obtain a small number of RecA domains on each dsDNA molecule that, in turn, allows extracting their dynamics from the dsDNA contour length variation. We measure the nucleation and growth rates of the individual domains in the presence of ATPγS and find that, on average, $k_{n}=\left(1.05\pm 0.05\right)\times 10^{-3}\;{\text{s}}^{-1}$ and $k_{g}=\left(5.8\pm 0.3\right)\times 10^{-4}\;{\text{s}}^{-1}$. Moreover, we find that domain growth is strongly asymmetric whereby the rate of growth is about 10 times larger for the fast front than for the slow one.

## Materials and Methods

### Experimental System

**FIGURE 1:**
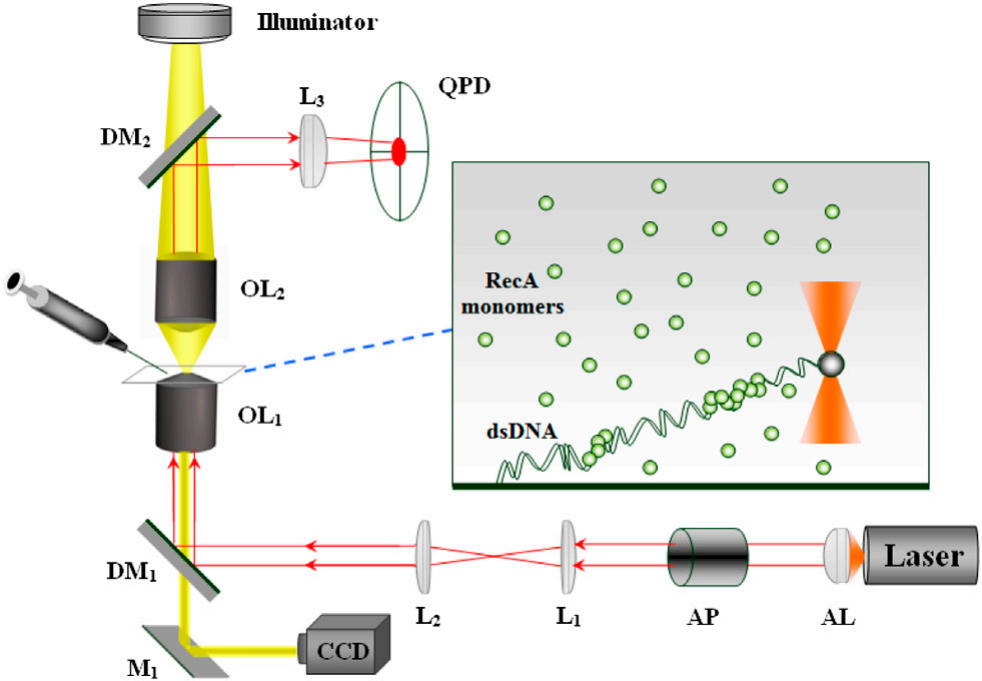
The optical system: Laser—single mode (TEM_00_) diode laser, AL—aspherical lens, AP—anamorphic prisms, L_1_, L_2_, L_3_—lenses, M_1_—mirror, DM_1_, DM_2_—dichoric mirrors, OL_1_, OL_2_—objectives. While OL_1_ focuses the laser beam forming the optical trap, OL_2_ collects the light scattered from the trapped bead that after reflection from DM_2_ reaches the QPD. The protein and the appropriate buffer are injected into the sample via a motorized syringe.

In our system, we use Optical Tweezers to apply and measure forces on individual dsDNA molecules. It consists of a near infrared laser beam (SDL 5422H1, 830 nm wavelength) focused through a 100X objective (Zeiss, 1.25 numerical aperture, oil immersion) together with a Quadrant Photodiode (QPD) that monitors the position of a trapped microbead (Figure 1). The power of the laser beam at the optical trap is about 30 mW. Single dsDNA molecules are attached at one end to the bottom cover glass and at the other end to a polystyrene microbead of 1.6 μm diameter (Polysciences). The experiment is performed in a flow cell that allows changing the biochemical composition of the sample. To avoid breaking the bead-dsDNA-cover glass constructs, we inject new solution at relatively low flow rates using a DC motor (Newport) to activate a syringe. Following the addition of RecA to the sample and its assembly on the dsDNA molecules, the dsDNA elongates by an extent proportional to the amount of bound protein. To monitor the change in the contour length of a particular dsDNA molecule, we extend the filament by applying an approximately constant force on the trapped bead (∼0.8 pN).

The force exerted by the dsDNA-RecA filament on the trapped bead, *F*, manifests as a shift in the bead position with respect to the center of the trap, Δ*x*. Correspondingly, the value of Δ*x* affects the distribution of the laser light scattered from the trapped bead. This variation in the distribution of the laser light transmitted through the sample is monitored by the QPD. Calibrating the QPD allows to deduce the value of Δ*x* from the voltage difference between the appropriate quadrants. Although the system allows a sampling rate of 20 KHz, we average the data down to rates in the 1–10 Hz range to reduce the effect of Brownian motion. The force is adjusted whenever it deviates from the 0.8 pN mark by an appropriate shift of the sample. Since the optical trap is at a fixed position, we move the sample to change the end-to-end distance of the DNA molecule, leading to a corresponding change in the force. A Peltier element together with a feedback control unit (PID) is used to maintain a constant temperature in the sample at 37.0 ± 0.1°C. It is attached to a copper ring that is in thermal contact with the microscope objective.

### Sample Preparation

The bead-dsDNA-glass construct is prepared using a low pH protocol for spontaneous binding (Allemand et al., 1997; Shivashankar et al., 1999). *λ*-DNA, 48.5 Kb, 16.5 μm (Promega) is incubated with the beads and PBS buffer at pH = 4 for 15 min to obtain the DNA-bead link. Next, the solution of DNA and beads is injected into the sample and after 2 h incubation we find that a certain fraction of the DNA molecules are tethered both to the glass bottom and to a microbead. Finally, we gently wash the sample to remove free beads and unbound DNA molecules. For a particular bead-dsDNA-glass construct, we verify that the bead is tethered by a single dsDNA molecule by measuring its force extension behavior and comparing it to the prediction of the Worm Like Chain (WLC) model (see Eq. 1) (Marko and Siggia, 1995). We then stretch the dsDNA and inject the protein together with the appropriate buffer solution into the sample cell. The final concentrations are as follows: RecA (9.33 μM), ATPγS (4.5 mM), MgCl_2_ (6.25 mM), DDT (6.25 mM), TrisHCl (18.75 mM), and the pH is 7.9. The pH and the protein and buffer concentrations were chosen such that the nucleation is sufficiently slow allowing to observe the dynamics due to individual nucleation events.

Although the low pH protocol for obtaining the bead-dsDNA-glass construct is not widely used in single molecule experiments, it is particularly straightforward allowing us to perform a relatively large number of experiments. On one hand, its main drawback is that it is by far less specific than, for example, the standard biotin:streptavidin tethers, leading to a fraction of bead-dsDNA-glass constructs where the dsDNA is attached at some internal site rather than at its end to either the glass or the bead. However, for our experimental approach, such constructs are equally suitable as those where the dsDNA is only tethered at its ends. To include the non-specific constructs in our experimental data, we measure the contour length of the dsDNA between tethering points for each construct using the WLC model (see Eq. 1) (Marko and Siggia, 1995). Moreover, we test that the length of the dsDNA is not affected by the increase in pH to 7.9 in preparation for the RecA reaction. On the other hand, the non-specific constructs allow obtaining data for different contour lengths of the naked dsDNA, an additional parameter that affects the polymerizarion dynamics of RecA on dsDNA.

### Measuring the Length of the dsDNA-RecA Complex

To monitor the length dynamics of the dsDNA-RecA complex during the polymerization process, we need to find the way it is related to the measured time dependence of the force exerted on the trapped bead. The equilibrium behavior of polymers under tension has been extensively studied (Smith et al., 1992; Perkins et al., 1995; Cluzel et al., 1996; Simmons et al., 1996; Wang et al., 1997). Using the WLC model, it was shown that the force, *F*, required to stretch the polymer to a certain end-to-end distance, *z*, is linear in the small *z* regime and rapidly grows as *z* approaches the contour length, *L* (Marko and Siggia, 1995). A good approximation to the exact F(z) is provided by the interpolation formula

$$F\left(z\right)=\frac{k_{B}T}{A}\left[\frac{z}{L}+\frac{1}{4{\left(1-z/L\right)}^{2}}-\frac{1}{4}\right]$$ (1)

where *A* is the persistence length, *T*, the temperature and $k_{B}$, the Boltzmann constant.

**FIGURE 2:**
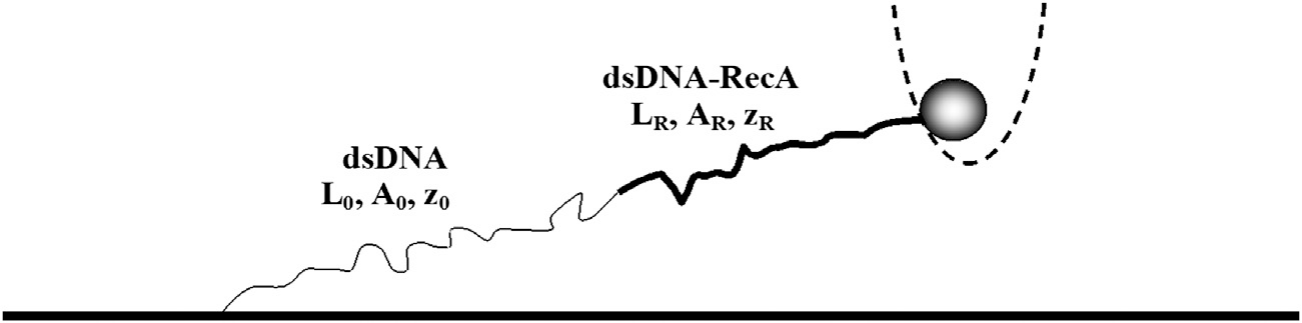
A dsDNA with a naked section (thin curve) and a single RecA domain (thick curve). Each segment of the molecule is characterized by its contour length, end-to-end distance and persistence length. In the experiment, we measure the force exerted by the dsDNA molecule on the trapped bead and the end-to-end distance of the entire molecule, *z*, as a function of time.

For naked dsDNA in solution of physiological ionic strength and pH≈7, it was found that the persistence length, $A_{0}$, is about 50 nm. The effect of the RecA binding on the force-extension behavior is twofold: 1. the RecA-dsDNA complex is longer that the naked DNA, leading to an increase in the contour length, L(t), as more RecA assembles on the dsDNA, 2. the RecA-dsDNA filament is significantly more rigid than the naked dsDNA molecule, $A_{R}\gg A_{0}$, where $A_{R}$ is the persistence length of RecA-dsDNA. At intermediate stages of the RecA-dsDNA filament assembly, several domains on the dsDNA are decorated with protein while the rest is naked. In the large extension regime, $F\gg k_{B}T/A$, one expects that the force-extension behavior of a partly decorated filament only depends on the decorated length fraction, *ϕ*, rather than on the specific partition into domains. Accordingly, the relation between force and extension in partially assembled filaments is equivalent to that of a filament with a single RecA-dsDNA domain starting at one of the ends of the dsDNA and the same value of *ϕ* (Figure 2). Moreover, in the $F\gg k_{B}T/A$ regime, we can neglect the first and last terms of Eq. 1 and characterize each of the decorated and the naked sections by their corresponding contour length, persistence length and end-to-end distance, $L_{R}$, $A_{R}$, $z_{R}$ and $L_{0}$, $A_{0}$, $z_{0}$, respectively (Figure 2). Clearly, $F=F_{R}=F_{0}$, $L\left(0\right)=\left(2/3\right)L_{R}+L_{0}$ and $z=z_{R}+z_{0}$, where *F*, $F_{R}$ and $F_{0}$ are the forces on the entire mixed filament, on the protein decorated domain and on the naked dsDNA part, respectively. Also, L(t) is the contour length and *z*, the end-to-end distance of the entire filament. Since in our experiment we measure F(t), L(0), Z(t) and $A_{R}$, these relations correspond to four equations with four unknowns, $L_{R}$, $L_{0}$, $z_{R}$ and $z_{0}$. Solving these equations provides an expression for the contour length of the protein decorated portion of the molecule

$$L_{R}=\frac{z-L\left(0\right)\left(1-C_{0}\right)}{\left(1/3\right)+\left(2/3\right)C_{0}-C_{R}}$$ (2)

where $C_{0}=\sqrt{k_{B}T/4FA_{0}}$ and $C_{R}=\sqrt{k_{B}T/4FA_{R}}$. Using the appropriate values for the persistence lengths, $A_{0}=50\;\text{nm}$ and $A_{R}\approx 1200\;\text{nm}$, together with the value of L(0) that is measured at the start of each experiment, we can use Eq. 2 to extract the contour length of the RecA-dsDNA domain from the measured values of F(t) and z(t). Since the total contour length is simply related to $L_{R}$, this allows obtaining the time dependence of the dsDNA decorated fraction, ϕ(t). While for the persistence length of the naked dsDNA, $A_{0}$, at the conditions of our experiment, we use the standard 50 nm value (Smith et al., 1992; Rocha et al., 2004), we have measured the value of $A_{R}$ for dsDNA molecules that were fully covered by RecA. Although we found some variation between the $A_{R}$ values obtained for different molecules, the 1,200 nm value represents a good approximation to the persistence length of the RecA-dsDNA complex whenever the RecA coverage is complete.

### Nucleation and Growth Model for the Fast nucleation Regime

In the regime where the rate of protein nucleation, $k_{n}$, is much larger than the rate of domain growth, $k_{g}$, we may assume that the number of domains, *N*, is a continuous variable. This allows obtaining a model that links the dynamics of N(t) with that of the DNA coverage, ϕ(t), via a set of two coupled differential equations (Avrami, 1939; Shivashankar et al., 1999)

$$\frac{dN}{dt}=k_{n}\left(1-\phi \right)-k_{g}\frac{N^{2}}{\left(1-\phi \right)}$$ (3a)

$$\frac{\text{d}\phi }{\text{d}t}=k_{g}N$$ (3b)

While the rate constants of Eqs. 3a,b depend on the number of RecA binding sites on the naked DNA, $L_{0}/a$, where *a* is the length of a RecA binding site (3 bp), these can be normalized to obtain the corresponding microscopic values, *n* and v, such that $k_{n}=nL_{0}/a$ and $k_{g}=av/L_{0}$. *n* is the nucleation rate density (per unit time per binding site) and v is the average growth velocity of individual domains. While the first term of Eq. 3a describes the creation of new nuclei at a rate proportional to the undecorated part of the dsDNA molecule, the second term depicts the reduction in the number of domains due to collisions between their fronts. The model assumes periodic boundary conditions on the dsDNA molecule (circular DNA). However, the model also assumes a large number of domains, N≫1, and since N has to be much larger than the total number of DNA base pairs, $L_{0}/a$, we are in the limit of large $L_{0}/a$ where boundary effects are negligible. In other words, the model of Eqs. 3a,b is equally accurate for both circular and linear DNA.

Equations. 3a,b can be easily solved leading to a sigmoidal behavior for the dynamics of the protein coverage

$$\phi \left(t\right)=1-\text{exp}\left(-\frac{1}{2}k_{n}k_{g}t^{2}\right)$$ (4)

Moreover, the model of Eqs. 3a,b can be generalized to include the case where the average rate of growth of the domains is asymmetric, such that, the growth rate in the 3′ to 5′ direction of the dsDNA is *r* times slower than that in the reverse direction. For this case

$$\phi \left(t\right)=1-\exp\left(-\frac{1}{2}k_{n}k_{g}^{\prime }t^{2}\left(r+1\right)\right)$$ (5)

where $k_{g}^{\prime }$ is the rate constant of the fast front such that the growth rate of the domain is $k_{g}=k_{g}^{\prime }\left(r+1\right)$. Note that, unlike $k_{g}$, $k_{g}^{\prime }$ represents the growth rate of individual fronts rather than that of entire domains. Since the behavior of the protein coverage in the fast nucleation regime (Eqs. 4, 5) depends only on the product of the reaction rates, $k_{n}k_{g}$, and the front propagation asymmetry factor, *r*, one cannot obtain each of these parameters separately by comparing between the model and the corresponding experimental measurements. Instead, to determine each of these parameters separately, the assembly of the protein on dsDNA needs to be analyzed in a regime where nucleation is not much faster that the growth rate.

### Nucleation and Growth Model for the Slow nucleation Regime

Another regime of the nucleation and growth process that can be described via an exactly solvable model is the limit of slow nucleation, $k_{g}/k_{n}\gg 1$. Here, we may assume that there is only one domain that grows to cover the entire dsDNA molecule. Since the location of the nucleation site for this domain can be anywhere along the dsDNA, the dynamics of the coverage, ϕ(t), is different for each realization. However, in all realizations, the coverage dynamics is bilinear. While, the first slope corresponds to the time before the first front reaches the end of the molecule, the second slope represents the time interval until the other front reaches the other end of the dsDNA

$$\dot{\varphi }\left(t\right)=\left(r+1\right)k_{g}^{\prime }-k_{g}^{\prime }\theta \left(tk_{g}^{\prime }-x\right)-k_{g}^{\prime }r\theta \left(rtk_{g}^{\prime }-\left(1-x\right)\right)$$ (6)

where *x* · *L* is the position of the nucleation event and θ(x) is the step function.

Averaging Eq. 6 over all possible realizations, gives the ensemble averaged coverage dynamics, $\bar{\phi }\left(t\right)$, (Turner, 2000)

$$\bar{\phi }\left(t\right)=\{\begin{array}{cc} k_{g}^{\prime }\left(1+r\right)t-\frac{1}{2}{k^{\prime }}_{g}^{2}t^{2}\left(1+r^{2}\right) & t<\frac{1}{k_{g}^{\prime }}\\ k_{g}^{\prime }rt-\frac{1}{2}{k^{\prime }}_{g}^{2}r^{2}t^{2}+\frac{1}{2} & \frac{1}{k_{g}^{\prime }}<t<\frac{1}{rk_{g}^{\prime }}\\ 1 & t>\frac{1}{rk_{g}^{\prime }} \end{array}$$ (7)

Unlike the behavior in the fast nucleation regime, here $\bar{\phi }\left(t\right)$ depends separately on the values of $k_{g}^{\prime }$ and *r* and does not depend on $k_{n}$. This can be used to determine both $k_{g}^{\prime }$ and *r* by fitting the behavior of Eq. 7 to the experimentally measured coverage dynamics in the low nucleation regime. Moreover, here asymmetric domain growth leads to a three step behavior for $\bar{\phi }\left(t\right)$ instead of the two step behavior of the symmetric case.

## Results

### Kinetics of RecA Protein Polymerization on Single dsDNA Molecules

#### Kinetics of the dsDNA-RecA Filament Length

**FIGURE 3:**
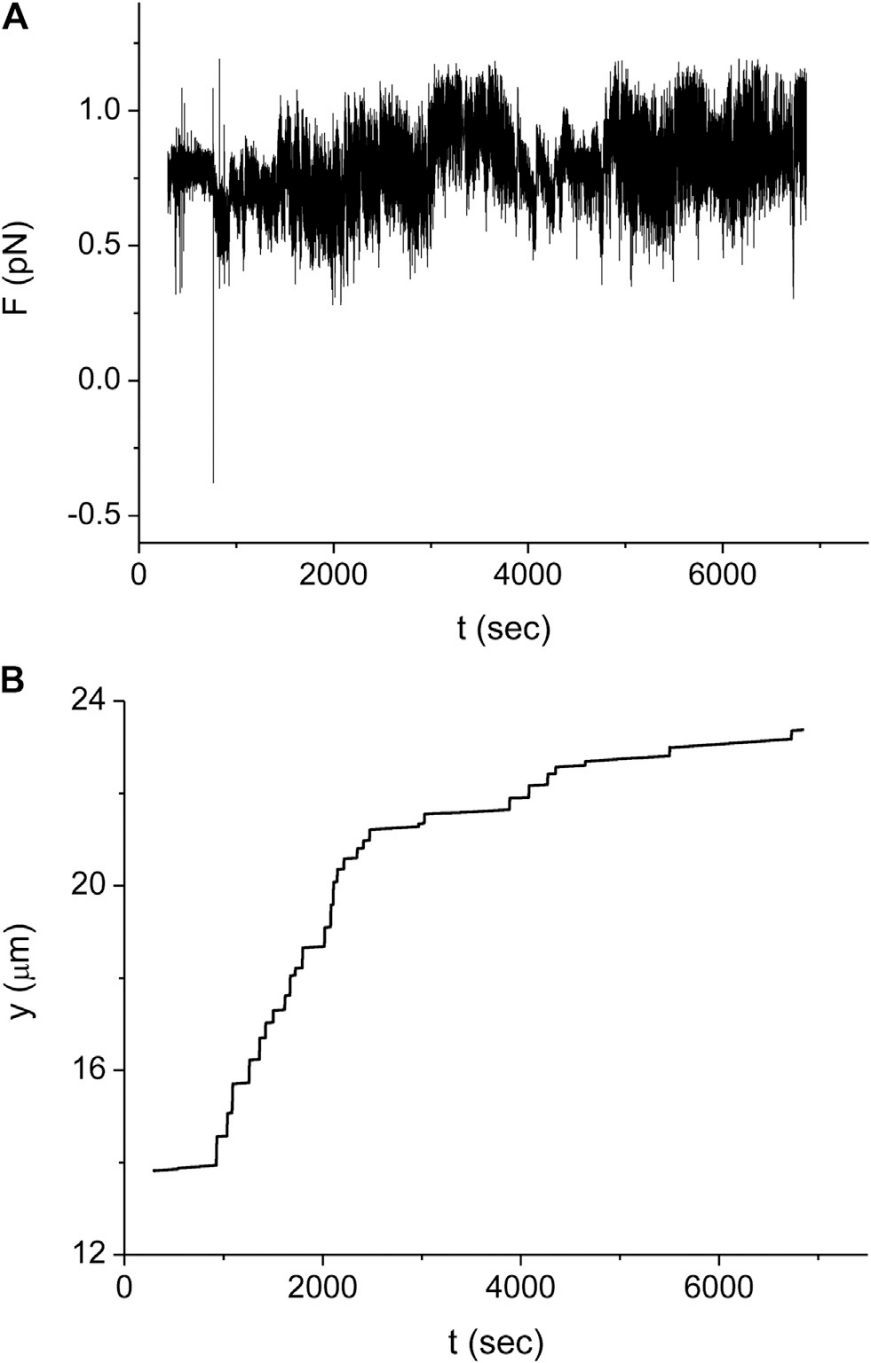
Typical experiment monitoring the RecA polymerization dynamics on a single dsDNA molecule. **(A)** Force as a function of time measured for a dsDNA molecule with *L* (0) = 16.4 μm.**(B)** Trap position relative to the point where the dsDNA molecule is tethered as a function of time for the same experiment as in **(A)**. Time is measured from the moment when we start to inject the protein into the sample. Since during protein injection and for a short period afterward there is noticeable fluid flow in the sample, no measurements were made during the corresponding time interval (about 300 s).

In our experimental setup, we measure the force exerted on the microbead by a single stretched dsDNA-RecA filament, *F*. For example, in Figure 3A the time trace of *F*, F(t), is shown for a molecule that is 16.4 μm long before the onset of RecA assembly. To perform the experiment under approximately constant force, we shift the position of the trap to maintain F(t) in a limited range around 0.8 pN, F(t)=0.8±0.1 pN. Figure 3B shows the variation in the distance between the trap position and the point where the dsDNA is attached to the coverslip, y(t), corresponding to the F(t) of Figure 3A.

**FIGURE 4:**
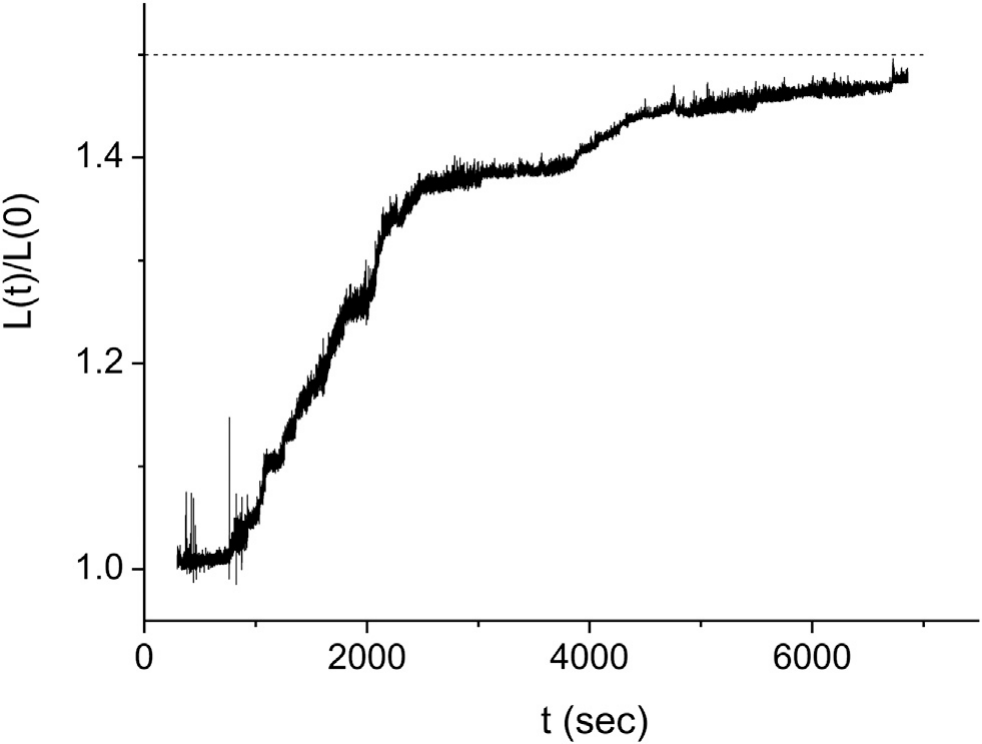
Kinetics of the DNA length as a function of time for the same experiment as in Figure 3 (full line). The dashed line indicates the expected saturation length corresponding to complete coverage of the dsDNA by RecA protein. The stepwise structure of the L(t) suggests that the domains grow significantly faster in one direction than in the other. Times when the slope of L(t) changes abruptly correspond either to a nucleus formation, the collision of two adjacent domains or the collision of a domain with an end of the DNA molecule.

In order to obtain the kinetics of the protein assembly on the dsDNA from the measurements of Figure 3, we use Eq. 2 where the value of the end to end distance, *z*, is approximated by the distance between the center of the optical trap and the dsDNA tethering point, *y* (see Figure 3B). The difference between these two quantities, *z* and *y*, is due to several, relatively small corrections that, moreover, almost cancel out. While the radius of the bead, 0.8 μm, and the displacement of the bead from the center of the trap due to the applied force, ∼0.2 μm, should be subtracted from the value of *y*, the effect due to the height of the trap above the bottom of the sample leads to an increase in the value of *z* relative to that of *y*. In our analysis, we assume that the effect of these corrections is below our experimental accuracy. In Figure 4 we show the resulting behavior of the RecA-dsDNA complex length as a function of time, L(t), for the same experiment as in Figure 3.

#### Kinetics of the Individual RecA-dsDNA Domains

The multiple step behavior of L(t) shown in Figure 4 corresponds to a regime that is intermediate between that of large number of nuclei, Eq. 5, and that of a single nucleus, Eq. 7. While Eq. 5 predicts a continuous sigmoidal growth of L(t), in the single nucleus limit, L(t) displays bilinear growth. In contrast, in the experiment of Figures 3, 4, the observed behavior is consistent with a scenario where a small number of domains compete to seize their share of the undecorated dsDNA molecule. Within this interpretation, the formation of a new domain would lead to a sudden increase in the rate of protein assembly corresponding to an increase in the slope of L(t). Moreover, whenever two domains collide or one domain collides with one of the ends of the dsDNA molecule, this would manifest as a sudden decrease in the slope of L(t). In what follows, we refer to such events as break points. Counting the number of break points in L(t) while an undecorated dsDNA becomes fully covered with RecA, *Q*, allows establishing the total number of protein domains, $N_{t}$, $N_{t}=\left(1/2\right)\left(Q-1\right)$. For example, in the case of Figure 4, the kinetics of L(t) can be interpreted as displaying five break points, *Q* = 5 (including the point where saturation occurs at L(t)=1.5L(0) that was not measured in Figure 4 and is assumed to take place at *t* > 6,850 s), corresponding to *N*_*t*_ = 2. Moreover, the slope of L(t) in between break points may be related to the rate of growth of the individual domain fronts present in the corresponding time interval. This relation also indicates that, for the case of the experiment shown in Figure 4. in the time interval between the first two break points, $t_{1}$ and $t_{2}$, the growth of L(t) is due to a single domain. Since at $t_{2}$ the growth rate of L(t) decreases significantly, we may infer that at this time one of the two fronts of this domain has stopped growing and that the remaining growth is due to the second front. Notably, within our interpretation of the data, the growth velocity of one front is about 12 times faster than the other. In what follows, we suggest that this strong asymmetry between the growth rates of the individual domain fronts represents a general feature in the RecA-dsDNA system in the presence of ATPγS.

**FIGURE 5:**
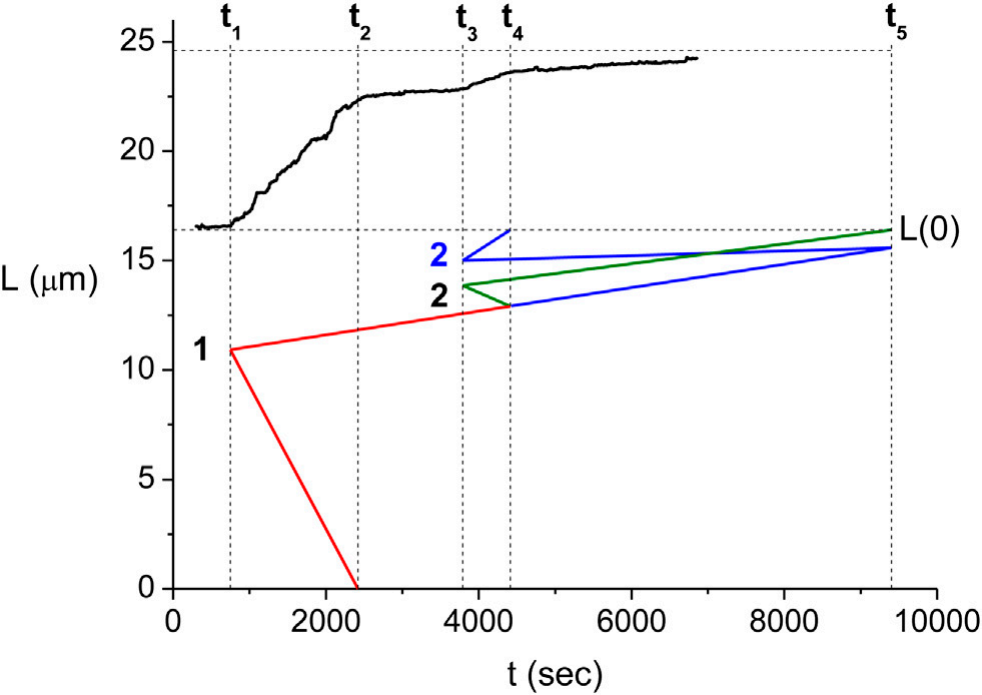
Relation between the domain kinetics and the measured L(t). The upper part of the figure shows the measured L(t) (black). Below the dashed line corresponding to the initial length, L(0), we show the front kinetics as obtained from the Eqs. 8a–f for the I-st scenario (red and green) and that for the II-nd scenario resulting from a similar set of equations (red and blue). Here, the position of the front is displayed using its location on the undecorated dsDNA. Note that the kinetics of the first domain (red) is identical for the two scenarios. This part of the domain kinetics is described by Eqs. 8a,b, e.

One may extend the interpretation relating the L(t) kinetics to that of the individual domains also to the regime where several domains coexist. To this end, we need to identify the collision scenario that is compatible with the observed growth pattern of L(t). Unlike in the single domain case, we find that for multiple domains there can be several scenarios leading to a particular growth pattern of the RecA-dsDNA. For the experiment of Figure 4, we identify times $t_{1}$ and $t_{3}$ as domain formation events and $t_{2}$, $t_{4}$ and $t_{5}$ as front collisions. However, time $t_{4}$ may correspond to the collision of the slow front of the first domain with the fast front of the second domain (black line in Figure 5) or to the collision of the fast front of the second domain with the end of the dsDNA molecule (blue line in Figure 5). We find that the multiple scenario behavior is not a special feature of the experiment of Figures 4, 5. On the contrary, the larger the number of growing protein domains on the dsDNA, the more decomposition scenarios will be consistent with a particular kinetics of the measured L(t).

Each of the kinetic scenarios consistent with a particular L(t) can be quantitatively analyzed to obtain the values of the parameters describing the domain trajectory on the undecorated dsDNA. Specifically, we need to determine the values of each of the front velocities, ${\text{v}}_{if}$ and ${\text{v}}_{is}$ (velocity of the fast and slow fronts of the *i*th domain, respectively), the position where the nucleation of the *i*th domain occurs, $x_{i}$, and the time of nucleation and domain collisions (break points), $t_{j}$, where both the index of the domains, *i*, and that of the break points, *j*, are ordered chronologically. To this end, we can relate between the individual domain parameters for a particular kinetic scenario and the time dependence of L(t) via a set of linear equations. For example, Eqs. 8a–f represent such relation for the case of the red and green scenario (the first scenario) of Figure 5

$${\text{v}}_{1s}+{\text{v}}_{1f}=2\upsilon_{1}$$ (8a)

$${\text{v}}_{1s}=2\upsilon_{2}$$ (8b)

$${\text{v}}_{1s}+{\text{v}}_{2s}+{\text{v}}_{2f}=2\upsilon_{3}$$ (8c)

$${\text{v}}_{2s}=2\upsilon_{4}$$ (8d)

$$x_{1}={\text{v}}_{1f}\left(t_{2}-t_{1}\right)$$ (8e)

$$x_{2}=x_{1}+{\text{v}}_{1s}\left(t_{4}-t_{1}\right)+{\text{v}}_{2f}\left(t_{4}-t_{3}\right)$$ (8f)

where $\upsilon_{j}$ is the slope of the best linear fit to the measured L(t) in the time interval between $t_{j}$ and $t_{j+1}$ (see Figure 5). While Eqs. 8a–d relate the front velocities of the individual domains to the rate of change in L(t) for each of the four time intervals between consecutive break points, Eqs. 8e,f determine the positions of the two nucleation sites from the detailed domain kinetics. Another set of equations similar to Equations (8a-f) describes the domain kinetics corresponding to the red and blue scenario (the second scenario) in Figure 5.

In the domain kinetics equations, e.g. those of Eqs. 8a–f, the values of $t_{j}$ and $\upsilon_{j}$, that are extracted directly from the time-dependence of L(t), play the role of parameters and $x_{i}$, ${\text{v}}_{if}$ and ${\text{v}}_{is}$ are the unknowns. However, we often find that for some of the scenarios compatible with the measured L(t) the solutions are unphysical, namely, some of the front velocities come out to be negative. Such negative values correspond to the disassembly of RecA proteins from the dsDNA which is negligible in our experiments since we use ATPγS instead of ATP. For example, for the second scenario of Figure 5 (red and blue), we obtain that ${\text{v}}_{2s}<0$ and therefore this scenario cannot take place. While in the case of the experiment of Figures 3–5 this mechanism leads to a single allowed scenario and thus, to unique domain kinetics, we find that, in general, there can be multiple scenarios where all front velocities are positive. As the number of protein nuclei on the dsDNA increases, it becomes more likely to find a larger number of different scenarios with only positive front velocities.

**FIGURE 6:**
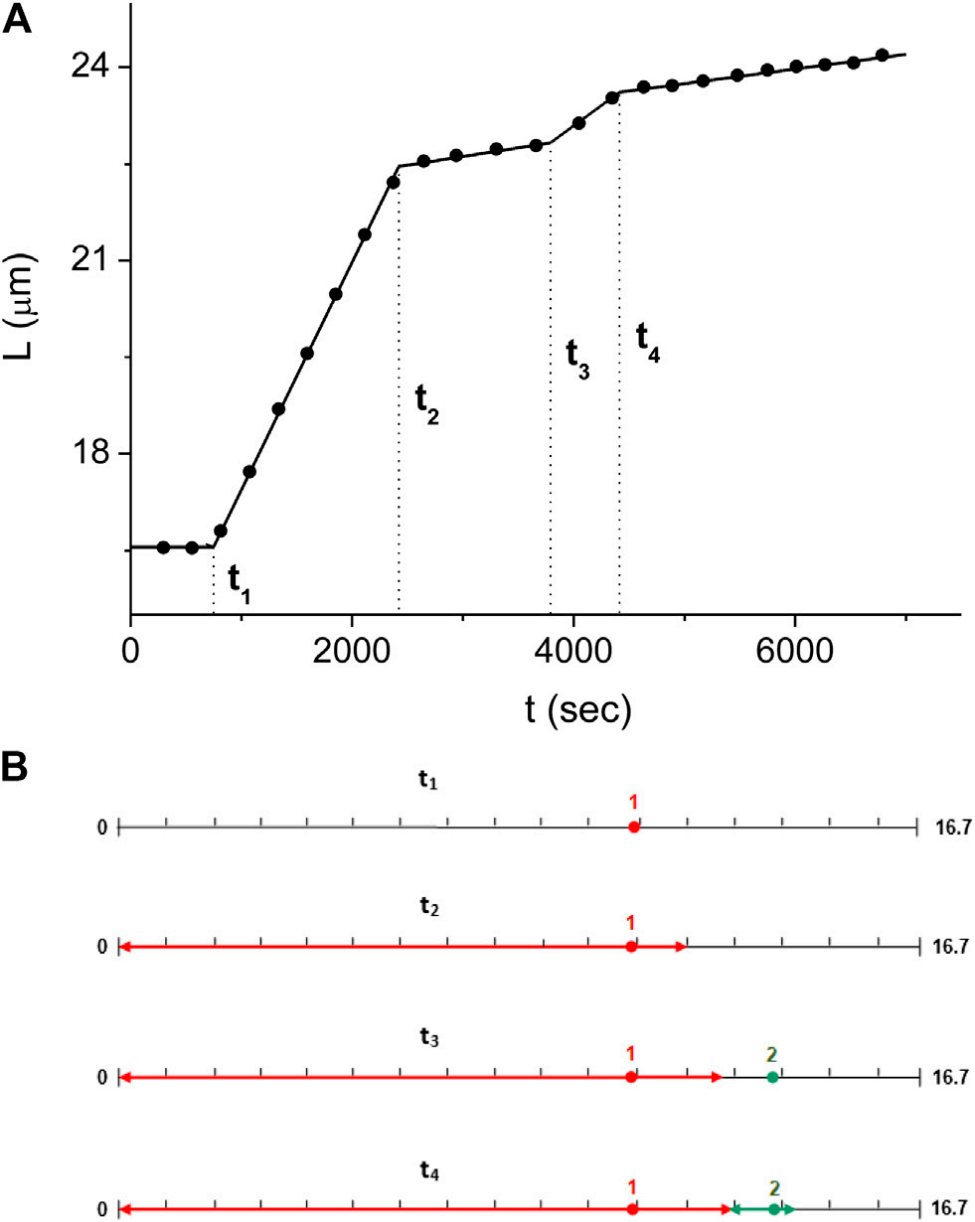
Domain kinetics corresponding to the solution of Eq. 8 **(A)** The dsDNA length kinetics, L(t), as obtained from the solution of Eq. 8 (line) is compared to the experimentally measured values of L(t) (full circles). To reduce fluctuations, each range of 2000 consecutive L(t) data points was averaged and the corresponding value is displayed at the mid-point of the respective time interval. **(B)** Illustration of the RecA domain distribution along the dsDNA at the four breakpoints, $t_{j}$. Following the color code of Figure 5, the first and second domains are displayed in red and green, respectively. The domain distribution is normalized to display positions with respect to the undecorated dsDNA. On this normalized scale, consecutive tick marks are 1 μm apart (except for the last two).

For the experiment of Figures 3–5, we obtain the values of $t_{j}$ and $\upsilon_{j}$ corresponding to the first scenario, fitting a bilinear function to L(t) for each $\left(t_{j-1},t_{j+1}\right)$ time domain. At first, the values of $t_{j}$ are directly estimated by inspection of the L(t) break points and used as initial values for an iterative computation. This approach converges to the best fitting piecewise linear function to L(t) after about four iterations. Solving Eq. 8 with the values of $t_{j}$ and $\upsilon_{j}$ obtained from the iterative fit of L(t), we obtain the locations of the nucleation sites, $x_{i}$, and the velocities of each of the domain fronts, ${\text{v}}_{if}$ and ${\text{v}}_{is}$, as illustrated in Figure 5. Moreover, we use the solution of Eq. 8 to simulate the L(t) corresponding to the domain kinetics for the first scenario (see Figure 6A) and find that the simulated L(t) is in good agreement with the experimental measurements. Figure 6B illustrates the distribution of the RecA domains at the times corresponding to each of the four break points.

### Statistical Analysis of the Domain Kinetics for RecA Polymerization on Single dsDNA

**FIGURE 7:**
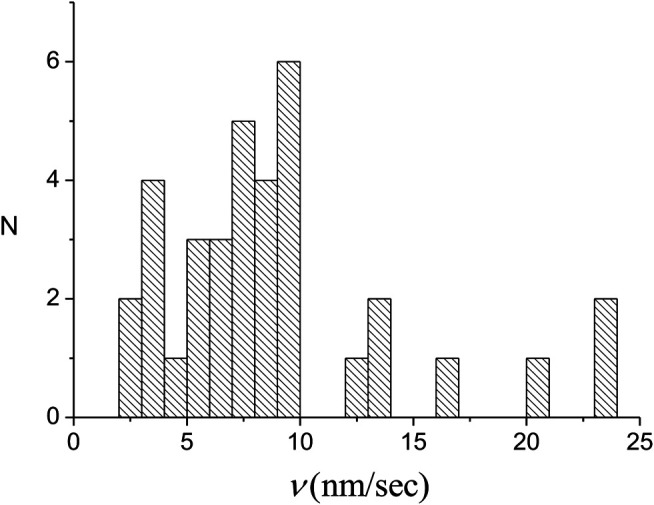
Distribution of the individual domain growth rates of 35 nuclei. For each domain the growth rate represents the sum of the rates for the slow and fast fronts.

We have performed a series of experiments under the same conditions as those leading to the results of Figures 3–5 (see Supplementary Section S1 for details). As one would expect, the L(t) kinetics that was measured is quite different from one experiment to another. However, in most experiments we observe the step-like structure reminiscent of that of Figure 4. Using the experiments where the number of nuclei is not too large, we repeated the analysis described in the previous section to obtain the full domain kinetics. Although in some of the experiments the domain kinetics was either incomplete or could be only partially analyzed, we also used such situations to extract the front velocities for some of the domains. For the latter, it is often difficult to distinguish between the velocities of the fast and slow fronts. Accordingly, we have used all available data to obtain the total velocities, $v_{i}={\text{v}}_{is}+{\text{v}}_{if}$, for 35 different domains. The distribution of these ${v_{i}}^{\prime }\text{s}$, $P\left(v_{i}\right)$, is displayed in Figure 7. The corresponding average, $\bar{v}=9\pm 1\;\text{RecA}\;{\text{sec}}^{-1}$, leads to a domain growth rate, ${\bar{k}}_{g}=a\bar{v}/{\bar{L}}_{0}=\left(7\pm 1\right)\cdot 10^{-4}\cdot {\text{sec}}^{-1}$.

The value of the average domain growth rate, ${\bar{k}}_{g}$, that was obtained from Figure 7 cannot be measured in the many nuclei regime of Eqs. 3–5, since the L(t) kinetics in this regime is fully determined by the $k_{n}k_{g}$ product. This suggests that in the few nuclei regime, where our study was performed, we can also establish the corresponding value of the average rate of nucleation, ${\bar{k}}_{n}$. To this end, we use the relation between the extent of undecorated dsDNA and the probability of a new nucleation event

$$\Delta p\left(t^{\prime }<t<t^{\prime }+\Delta t\right)=nL_{free}\left(t\right)\Delta t$$ (9)

where $\Delta p\left(t^{\prime }<t<t^{\prime }+\Delta t\right)$ is the probability of forming a new nucleus in the $\left(t^{\prime },t^{\prime }+\Delta t\right)$ time interval, Δt represents an infinitesimal time step and $L_{free}\left(t\right)$ is the part of dsDNA not covered by protein at time *t*. Integrating Eq. 9 between one nucleation event at $t_{i}$ and the next at $t_{i+1}$, the left side becomes equal to 1 and on the right side we obtain the integral of $L_{free}\left(t\right)$ between $t_{i}$ and $t_{i+1}$ times *n*

$$1=n\int_{t_{i}}^{t_{i+1}}L_{free}\left(t\right)\text{d}t$$ (10)

Moreover, the relation of Eq. 10 can be expanded to include the first *N* nucleation events, namely,

$$N=n\int_{t_{0}}^{t_{N}}L_{free}\left(t\right)\text{d}t=nB_{N}$$ (11)

where $t_{0}$ is the time when the protein was added to the sample, *B*_*N*_ denotes the time integral of $L_{free}\left(t\right)$ from $t_{0}$ to the time when the *N*th nucleus is formed, $t_{N}$, and $L_{free}\left(t\right)$ is directly related to the measured L(t), $L_{free}\left(t\right)=3L\left(0\right)-2L\left(t\right)$.

**FIGURE 8:**
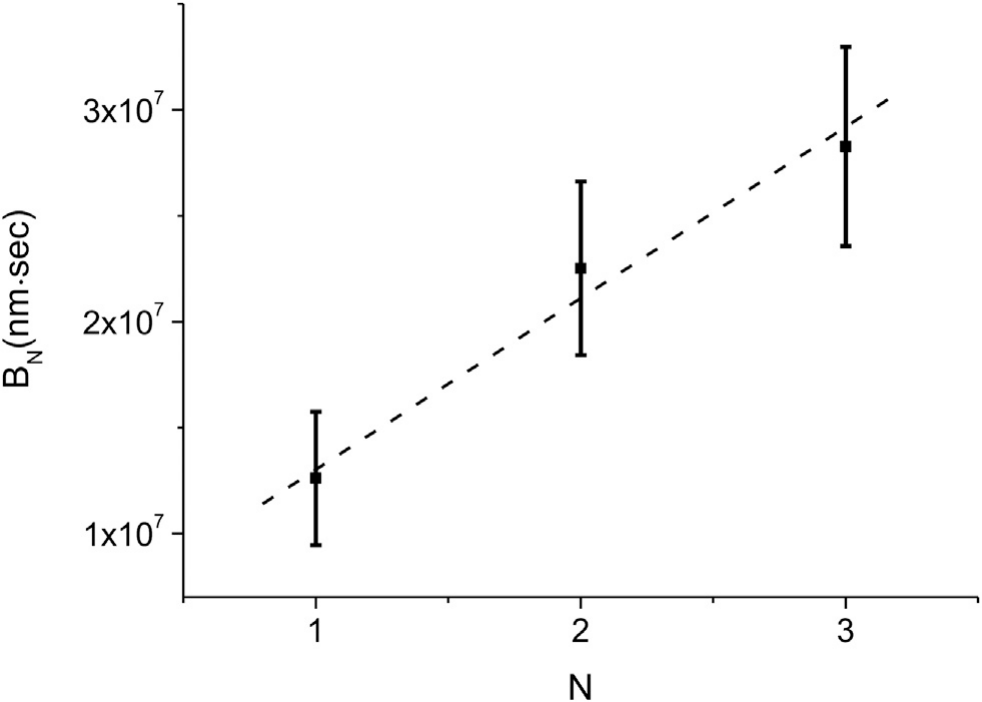
Experimental measurement of the average nucleation density rate, $\bar{n}$. The values of *B*_1_, *B*_2_, and *B*_3_ were obtained from the L(t) measured in the different experiments that were performed under identical conditions. The corresponding averages, ${\bar{B}}_{N}$, and their standard deviations are shown as data with error bars (squares). The dashed line represents the best linear fit to ${\bar{B}}_{N}$. From its slope we obtain $\bar{n}=\left(13\pm 4\right)\cdot 10^{-8}\text{binding}\;{\text{site}}^{-1}\cdot {\text{sec}}^{-1}$ and ${\bar{k}}_{n}=\left(1.5\pm 0.5\right)\cdot 10^{-3}\cdot \sec^{-1}$.

Equation 11 allows us to estimate the average nucleation rate density, $\bar{n}$. This is achieved by computing the values of *B*_*N*_ for each of the different experiments and obtaining the corresponding averages, ${\bar{B}}_{N}$. Then, according to Eq. 11, the slope of the plot of ${\bar{B}}_{N}$ vs. *N* is ${\bar{n}}^{-1}$ (see Figure 8). The best linear fit to the ${\bar{B}}_{N}$ vs. *N* data shown in Figure 8 leads to $\bar{n}=\left(13\pm 4\right)10^{-8}\;\text{binding}\;{\text{site}}^{-1}\cdot {\text{sec}}^{-1}$, which, in turn, corresponds to an average nucleation rate, ${\bar{k}}_{n}=\left(1.5\pm 0.5\right)\cdot 10^{-3}\cdot \sec^{-1}$.

For three of our experiments, the domain kinetics inferred from our model allowed to obtain the velocity of each of the individual fronts. In these experiments, the L(t) kinetics were complete, had well separated nucleation times and their decomposition was unambiguous. Of these three experiments, one had two nuclei and its full analysis was presented in the previous section. The other two experiments had one and three nuclei, respectively, leading to a total of six nuclei for which we can estimate, *r*, the velocity ratio between the slow and the fast fronts, $\bar{r}=0.25\pm 0.08$.

While in the few nuclei regime where we have performed our experiments we can separately measure the average values of the nucleation and growth rates, ${\bar{k}}_{n}$ and ${\bar{k}}_{g}$, the theoretical description of the protein assembly process in this regime cannot be described by the analytical models presented in the Materials and Methods section, Eqs. 3–7. Instead, we need to use a nucleation and growth model that describes the formation of a few nuclei at random positions along the dsDNA and the dynamics whereby the emerging protein domains expand to cover the entire dsDNA molecule. We use numerical simulation to predict the average domain kinetics for our model and compare the outcome to the average experimental L(t). This comparison also provides an alternative method to measure the values of the average nucleation and growth rates, ${\bar{k}}_{n}$ and ${\bar{k}}_{g}$.

### Nucleation and Growth Model for the Few Nuclei Regime

**FIGURE 9:**
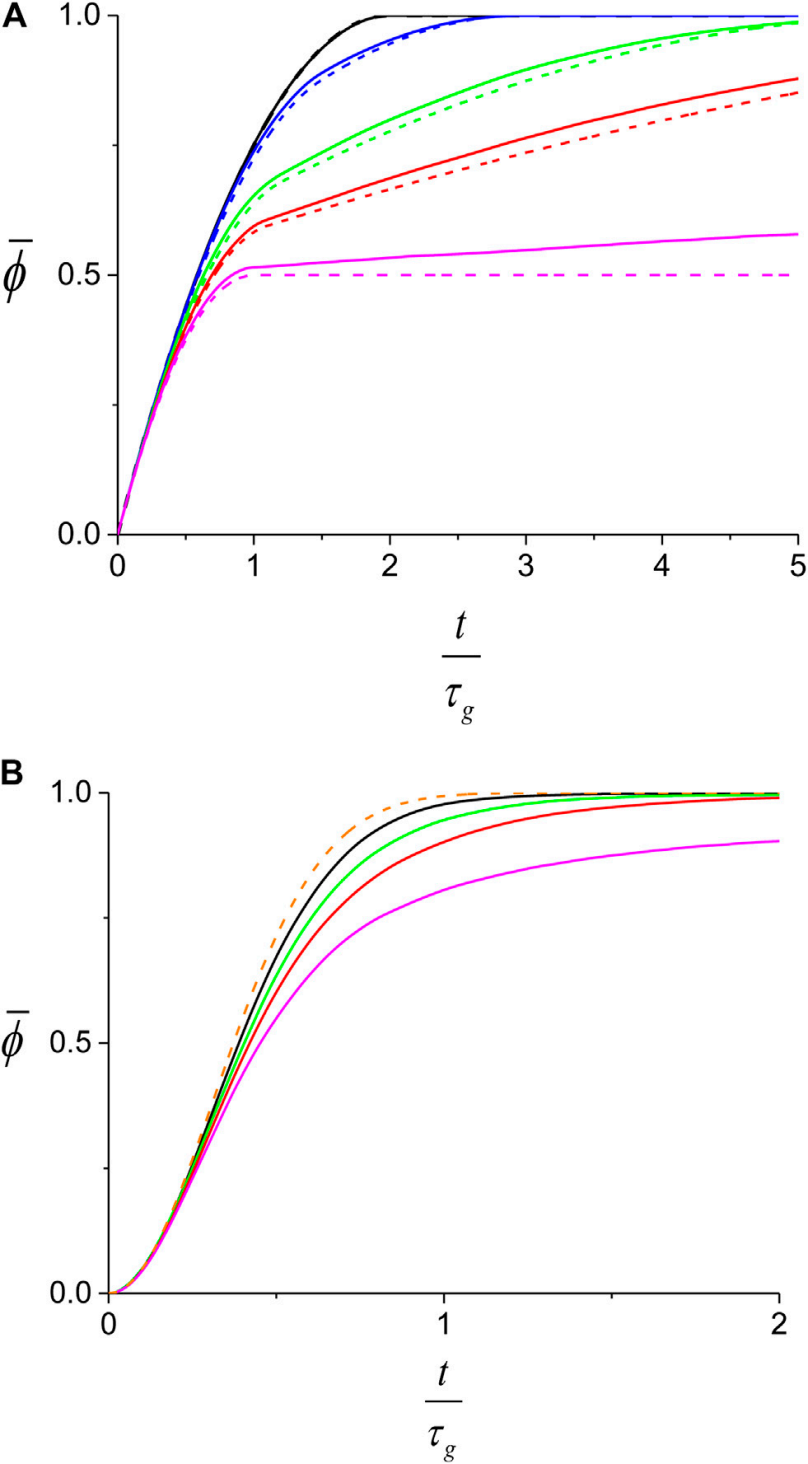
Theoretical prediction for the average protein coverage on dsDNA. While the full lines are obtained from the Monte-Carlo simulation, the dashed lines correspond to the analytical behavior described by Eqs. 5, 7. **(A)** slow nucleation regime, $\left(k_{g}/k_{n}\right)=10$, $k_{n}=10^{-4}\cdot \sec^{-1}$, for different values of *r*: *r* = 0 (magenta), *r* = 0.1 (red), *r* = 0.2 (green), *r* = 0.5 (blue) and *r* = 1 (black). *t* = 0 corresponds to the first nucleation event. For each case, we also show the corresponding prediction of Eq. 7 (dashed). **(B)** fast nucleation regime, $\left(k_{g}/k_{n}\right)=0.1$, $k_{n}=10^{-2}\cdot \sec^{-1}$, for same values of *r* as in **(A)** except for *r* = 0.5 which is too close to the *r* = 1 curve. The prediction of Eq. 5 is also shown (orange dashed). Unlike in **(A)**, here the first nucleation event will occur in the $\left(t_{1},t_{1}+\text{d}t\right)$ time interval with probability $t_{1}nL\left(0\right)\text{d}t$.

In the intermediate regime, where $k_{n}$ and $k_{g}$ are of the same order, we describe the nucleation and growth of the RecA on dsDNA using a Monte Carlo type model (Turner, 2000). It consists of two components: 1. nucleation of a protein monomer at a random position, *x*_*i*_, along the (0, *L*) interval, and 2. growth of the *i*th domain starting at *x*_*i*_ and growing with rate $k_{g}^{\prime }$ at one end and $rk_{g}^{'}$, 0≤r≤1, at the other. The time step for the simulation, δt, was set to be 0.5 s, sufficiently small to ensure that the resulting kinetics does not depend on δt. At each time step, a nucleation event will occur with probability $k_{n}\left(1-\phi \right)\delta t$ at a random location along the undecorated part of the dsDNA. In addition, each of the existing domains will grow by $k_{g}^{\prime }\delta t$ at its fast front and by $k_{g}^{\prime }r\delta t$ at its slow front. The side of the *i*th domain corresponding to the fast front is randomly chosen at $t_{i}$, its nucleation time. The simulation is stopped when the protein covers the entire dsDNA molecule, ϕ=1, except for the case when *r* = 0 where full coverage occurs after very long time (Figure 9). To obtain the average kinetics of the protein coverage, $\bar{\phi }\left(t\right)$, we repeat the simulation *M* times and average ϕ(t) over all the runs

$$\bar{\phi }\left(t\right)=\frac{1}{M}\sum_{l=1}^{M}\phi_{l}\left(t\right)$$ (12)

The fluctuations of $\phi_{l}\left(t\right)$ between one run and another are quantified by the corresponding standard deviation, σ(t),

$$\sigma^{2}\left(t\right)=\frac{1}{M}\sum_{l=1}^{M}{\left(\phi_{l}\left(t\right)-\bar{\phi }\left(t\right)\right)}^{2}$$ (13)

To obtain the behavior of $\bar{\phi }\left(t\right)$ in the different parameter regimes, we have used a relatively small ensemble, $M=10^{3}$. At this level of averaging, the corresponding error of $\bar{\phi }\left(t\right)$, $\sigma \left(t\right)/\sqrt{M}$, is too small to be graphically resolved (Figure 9).

In Figure 9 we show the behavior of the average coverage, $\bar{\phi }\left(t\right)$, for both the slow and fast nucleation regimes and compare the numerical results to the corresponding theoretical predictions of Eqs 5, 7. In both these equations, time appears multiplied by the domain growth rate, $k_{g}=\left(1+r\right)k_{g}^{\prime }$, that sets the time scale for the kinetics in the different regimes. Therefore, in order to compare the kinetics of the nucleation and growth process at different values of the parameters it is necessary to normalize the time by the corresponding time scale $\tau_{g}=k_{g}^{-1}$. Moreover, we found that the $\bar{\phi }\left(t\right)$ kinetics for a particular growth asymmetry, *r*, only depends on the $k_{g}/k_{n}$ ratio and not on $k_{g}$ and $k_{n}$ separately (not shown). To examine the effect of the growth asymmetry on the nucleation and growth process for each regime, we fixed the nucleation rate, $k_{n}$, and varied the values of *r* and $k_{g}^{\prime }$, such that $k_{g}$ remains constant (Figure 9).

For the slow nucleation regime, the larger the growth asymmetry, smaller *r*, the slower will be the process of decorating the dsDNA on the side of the slow front of the first domain. Consequently, for small *r*, the $\bar{\phi }\left(t/\tau_{g}\right)$ increases at a slower rate and the RecA proteins are less efficient in covering the dsDNA (Figure 9A). Moreover, while Eq. 7 only describes the growth kinetics for a single domain, at small *r* the undecorated fraction of the dsDNA at a particular time is larger, leading to a larger probability for a second nucleation event to occur. The contribution of the *i* > 1 domains to $\bar{\phi }\left(t/\tau_{g}\right)$ leads to a growing discrepancy between the prediction of Eq. 7 and the results of the simulation as *r* decreases .

The lower efficiency of protein coverage for asymmetric growth is also found in the fast nucleation regime (Figure 9B) and can be ascribed to a mechanism similar to the one presented in the previous paragraph. When the number of domains is not too large, asymmetric growth can lead to persisting, relatively large undecorated sections between two slow growing fronts. The process of protein assembly in such regions remains inefficient until a new nucleation event occurs there. In contrast, the prediction of Eq. 9 corresponds to the limit where the number of domains is infinite and therefore, the growth asymmetry has no effect on $\bar{\phi }\left(t/\tau_{g}\right)$. We also found that the discrepancy between the behavior of $\bar{\phi }\left(t/\tau_{g}\right)$ as obtained from the simulation and that of Eq. 9 diminishes for lower values of $k_{g}/k_{n}$, due to the larger number of domains present at any particular $t/\tau_{g}$ (not shown).

**FIGURE 10:**
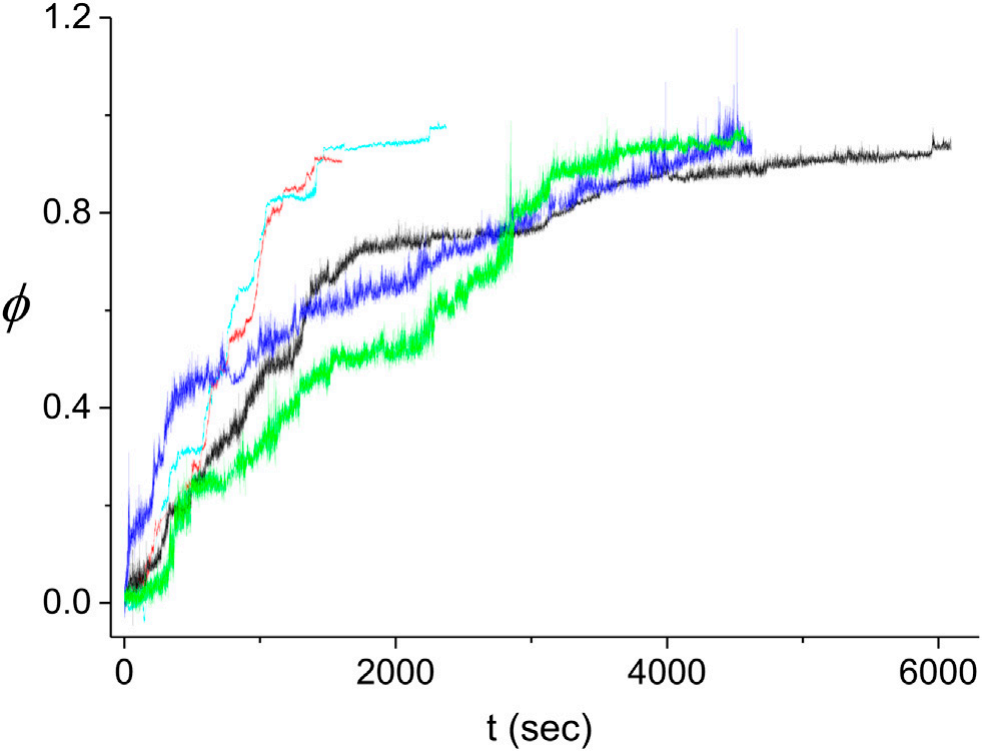
Kinetics of the decorated length fraction of the dsDNA. The curves correspond to five different experiments performed under identical conditions (see text). The black curve is the same as in Figures 4–6. The difference in the fluctuations in the light blue and red curves as compared to those in the other three curves is due to the different extent of averaging of the raw data from the QPD (sampled at 20 kHz). To obtain a similar accuracy in the decomposition of L(t) into linear segments for short time experiments, ∼2000 s, and long time experiments, ∼5,000 s, we had to use more data smoothing for the former than for the latter.

As discussed in the previous section, our experiments were performed in the few nuclei regime where neither Eq. 5 nor Eq. 7 are valid. Instead, we can use the Monte Carlo model described above to predict the expected behavior of $\bar{\phi }\left(t\right)$. In Figure 10, we show the kinetics of the RecA decorated fraction on the dsDNA as obtained in five different experiments performed under identical conditions (pH = 7.9, T = 37.0 ± 0.1°C, F = 0.8 ± 0.1 pN, [RecA] = 9.33 μM, [ATPγS] = 4.5 mM, [MgCl_2_] = 6.25 mM, [DDT] = 6.25 mM and [TrisHCl] = 18.75 mM). For our analysis, we selected only experiments that are complete, namely, those that reach ϕ≃1 (see Supplementary Section S1). Moreover, since we cannot determine the diffusion time of the proteins between the injection point to the neighborhood of the dsDNA molecule that is being examined, *t* = 0 was set to the time of the first nucleation event. The stepwise nature of ϕ(t) is more pronounced in some of the experiments of Figure 10 (e.g. black and cyan curves) than in others (e.g. blue curve) and the time scale of the kinetics varies significantly between experiments.

**FIGURE 11:**
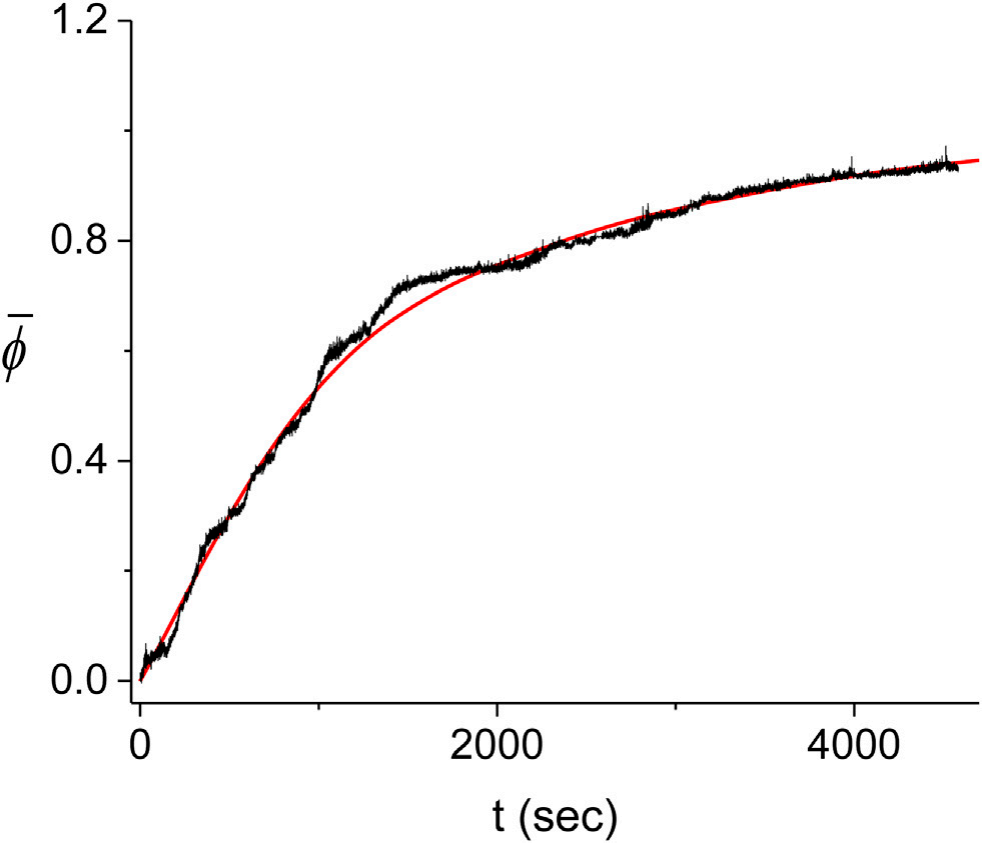
Average kinetics of the decorated length fraction of the dsDNA. The average of the five experiments shown in Figure 10 (black) is compared with the corresponding best fitting result from the Monte Carlo model (red). The best fit was obtained for r=0.10±0.05, $k_{n}=\left(1.05\pm 0.05\right)\cdot 10^{-3}\cdot \sec^{-1}$ and $k_{g}=\left(5.8\pm 0.3\right)\cdot 10^{-4}\cdot \sec^{-1}$.

The large variability between the experimental kinetics obtained in individual experiments indicates that these cannot be individually described by our Monte Carlo model. Instead, we expect that the model should be able to reproduce the average experimental kinetics, $\bar{\phi }\left(t\right)$. Accordingly, we average the time traces of Figure 10 and compare the resulting curve to the prediction of the model. The comparison consists of finding the best fitting theoretical kinetics, $\bar{\phi }\left(t\right)$, to the experimental average as a function of the model parameters $r,k_{n},k_{g}^{\prime }$ (see Figure 11). To obtain the best fit, we minimize the corresponding $\chi^{2}$-function which includes the errors of both the theoretical and the experimental $\bar{\phi }\left(t\right)$ kinetics. These errors are obtained from the fluctuations between the individual time traces, ϕ(t), that are used to compute the average kinetics, $\bar{\phi }\left(t\right)$. We find that the value of the $\chi^{2}$ function manifests a large variability whenever the averaging of the Monte Carlo model is insufficient. To determine the value of the $\chi^{2}$ function with enough accuracy, ∼1%, we have to use $M=10^{5}$, mainly in the parameter range around the minimum, which is a relatively heavy computation. Standard fitting routines were unable to obtain the parameter values corresponding to the minimal $\chi^{2}$, leading to either local or spurious minima. Instead, our search for the minimum of the $\chi^{2}$ function is performed by means of a three dimensional scan in the 3 parameter space, $r,k_{n},k_{g}^{\prime }$, whereby we average more (larger *M*) in the regions where the value of $\chi^{2}$ is smaller. This is an iterative method using first limited averaging to map the $r,k_{n},k_{g}^{\prime }$ space, $M=10^{3}$, with a ∼10% accuracy in $\chi^{2}$. This is followed by a two step increase in *M*, $M=10^{4}$ and $M=10^{5}$, within regions of the parameter space where the $\chi^{2}$ values obtained in the previous step were below an appropriate threshold. This approach allows us to zoom in on the region of the minimal $\chi^{2}$. The step for the scan is chosen such that the error in $\chi^{2}$ at its minimum, ∼1%, is smaller than the difference between the value of the computed $\chi^{2}$ at the minimum, $\chi^{2}=4318$, and that at any of the neighboring points on the scan grid.

For simplicity, we also use the values of the scan steps to represent the errors of the best fitting parameters. These errors are therefore overestimated and should be regarded as upper bounds and of the same order of magnitude as the exact values. We find that the best fit to the experimental $\bar{\phi }\left(t\right)$ is obtained for $\bar{r}=0.10\pm 0.05$, ${\bar{k}}_{n}=\left(1.05\pm 0.05\right)\cdot 10^{-3}\cdot \sec^{-1}$ and ${\bar{k}}_{}^{g}=\left(5.8\pm 0.3\right)\cdot 10^{-4}\cdot \sec^{-1}$. The corresponding values for the average nucleation rate density, $\bar{n}$, and the average domain growth velocity, $\bar{v}$, are $\bar{n}=\left(9.8\pm 0.5\right)\cdot 10^{-8}\cdot \text{binding}\;{\text{site}}^{-1}\cdot \sec^{-1}$ and v¯=6.2±0.3 RecA⋅sec−1, similar to the results obtained in the previous section from the analysis of the individual domains (see also Table 1). Due to the variability in the length of the dsDNA’s in our experiments, only the values of the molecular parameters, $\bar{n}$ and $\bar{v}$, that were obtained using the methods of this section and those of the previous one, namely, the Monte Carlo model analysis and the domain statistics, respectively, should be equivalent.

**TABLE 1:** The parameters that describe the nucleation and growth process in our experiment as obtained by each our two methods: averaging over the kinetics of individual domains as obtained from the decomposition of the L(t) (first column) and fitting the $\bar{\phi }\left(t\right)$ computed from the Monte-Carlo model to the one measured in experiments (second column).

	Kinetics of individual domains	Comparing average $\phi {\left(t\right)}^{\prime }\text{s}$
$\left({\bar{L}}_{0}/a\right)\left(\text{binding sites}\right)$	11,800	10,700
${\bar{k}}_{n}\left(\sec^{-1}\right)$	$\left(1.5\pm 0.5\right)\times 10^{-3}$	$\left(1.05\pm 0.05\right)\times 10^{-3}$
${\bar{k}}_{g}\left(\sec^{-1}\right)$	$\left(7\pm 1\right)\cdot 10^{-4}$	$\left(5.8\pm 0.3\right)\times 10^{-4}$
$\bar{r}$	0.25±0.08	0.10±0.05
$\bar{v}\left(\text{RecA}{\cdot \sec}^{-1}\right)$	9±1	6.2±0.3
$\bar{n}\left(\text{binding}\;{\text{site}}^{-1}\cdot \sec^{-1}\right)$	$\left(13\pm 4\right)\cdot 10^{-8}$	$\left(9.8\pm 0.5\right)\cdot 10^{-8}$
$\Delta E_{coop}\left(k_{B}T\right)$	17.8±0.4	17.9±0.3
$\Delta E_{asym}\left(k_{B}T\right)$	1.4±0.3	2.3±0.5

## Discussion

Measuring the force exerted on microbeads with accuracy better than 0.1 pN, we have monitored the polymerization of the RecA protein on individual dsDNA molecules in the presence of ATPγS. Since the RecA-dsDNA complex is about 1.5 times longer than the naked dsDNA, the polymerization process leads to a gradual increase in the contour length of the dsDNA allowing us to obtain the kinetics of the protein coverage on a particular dsDNA molecule. In the presence of ATPγS, the protein coverage is essentially irreversible (Lindsley and Cox, 1989). We show that a model which assumes a nucleation and growth mechanism exhibiting a small number of nuclei in the parameter range used in our experiments yields predictions that are consistent with our experimental data. In this regime, we can decompose the global coverage kinetics to infer the kinetics for each of the growth fronts that develop on the two sides of a nucleus. We therefore obtain the distribution of front velocities and find that each domain grows asymmetrically with a fast front that has, on average, a velocity about 10 times larger than that of the corresponding slow front.

In the few nuclei regime, the kinetics of the protein coverage on a single dsDNA, ϕ(t), manifests as a stepwise graph (Figure 4). This behavior is unlike that of the multiple nuclei limit, $\left(k_{g}/k_{n}\right)\gg 1$, of Eq. 5 or the single nucleus case, $\left(k_{g}/k_{n}\right)\gg 1$, of Eq. 7. Whenever a new domain is created or two fronts collide, L(t) displays a break point, namely, a sudden change in slope. The number of break points, *Q*, is related to the number of nuclei, $N_{t}$, $N_{t}=\left(1/2\right)\left(Q-1\right)$. Moreover, an increase in the slope of L(t) indicates a nucleation event while a decrease corresponds to a collision between two fronts or that of a front with an end of the molecule. In between consecutive break points, the slope of L(t) corresponds to the sum of the active fast and slow front velocities, ${\text{v}}_{if}$ and ${\text{v}}_{is}$, and we use this relation to obtain the values of ${\text{v}}_{if}$ and ${\text{v}}_{is}$. Averaging the corresponding domain velocities, $v_{i}$, $v_{i}={\text{v}}_{is}+{\text{v}}_{if}$, obtained from 11 different experiments, leads to the average domain growth rate, ${\bar{k}}_{g}=\left(a\bar{v}/{\bar{L}}_{0}\right)=\left(7\pm 1\right)\cdot 10^{-4}\cdot {\text{sec}}^{-1}$. Similarly, the analysis of the nucleation times allows to deduce the average $k_{n}$, ${\bar{k}}_{n}=\left(\bar{n}\cdot {\bar{L}}_{0}/a\right)=\left(1.5\pm 0.5\right)\cdot 10^{-3}\cdot \sec^{-1}$ (Eq. 11 and Figure 8). For only three of our 11 experiments the decomposition of L(t) allowed to uniquely determine each of the slow front and fast front velocities of all domains, ${\text{v}}_{is}$ and ${\text{v}}_{if}$, and their corresponding ratio, the growth asymmetry coefficient *r*. Averaging over all the domains that determined the L(t) kinetics in these three experiments we obtain $\bar{r}=0.25\pm 0.08$. Since only six domains are included in the average, a rather small number, this value of $\bar{r}$ should be regarded as a relatively rough estimate for the growth asymmetry coefficient.

An alternative approach that allows to obtain the rate constants relies on modeling the nucleation and growth process in the intermediate regime where $k_{n}$ and $k_{g}$ are of the same order. Using a Monte-Carlo model that depends on three parameters, $k_{n}$, $k_{g}^{\prime }$ and *r*, we compute the corresponding average coverage kinetics, $\bar{\phi }\left(t\right)$, and fit it to the experimentally measured average $\bar{\phi }\left(t\right)$ as obtained from five different experiments (Figures 10, 11). The best fitting theoretical $\bar{\phi }\left(t\right)$ to the experimental $\bar{\phi }\left(t\right)$ corresponds to ${\bar{k}}_{n}=\left(\bar{n}\cdot {\bar{L}}_{0}/a\right)=\left(1.05\pm 0.05\right)\times 10^{-3}\cdot \sec^{-1}$, ${\bar{k}}_{g}=\left(a\bar{\nu }/{\bar{L}}_{0}\right)=\left(5.8\pm 0.3\right)\times 10^{-4}\cdot \sec^{-1}$ and $\bar{r}=0.10\pm 0.05$. Moreover, the value of the $\chi^{2}$ at its minimum is 4,318, leading to a confidence level, *CL*, that, for all practical purposes, equals unity. In other words, we find very good agreement between the prediction of the Monte-Carlo model and our experimental results within the accuracy of our experiments. The high quality of the agreement is illustrated in Figure 11.

Since in our search for a low nucleation regime we used particular experimental parameters that are different from those of other studies, it is difficult to compare our results to those of previous studies. For example, our ATPγS concentration is 4.5 times larger than that used in Ref. (Shivashankar et al., 1999), the RecA concentration is almost the same, the other chemicals are at about a third of their concentration and our pH is 7.9 while theirs is 6.8. As one would expect, in the corresponding ATPγS experiment presented in Ref. (Shivashankar et al., 1999) (Figure 5B), Shivashankar et al. estimate to have 6 domains and the overall length dynamics is well approximated by the multiple domain dynamics of Eq. 3. The value of the product between the DNA length independent nucleation and growth constants, nν, that they obtain is 0.044 min^−2^, much larger than in our measurements, 0.002 min^−2^. The low value in our experiments is mostly due to a significantly lower nucleation rate.

### On the Nucleation and Growth Rate Constants

Although RecA and dsDNA are complex biomolecules, we may obtain further insight on the way they assemble in the presence of ATPγS by comparing this process to a simple bimolecular reaction of the type

A + B→C (14)

where the RecA-ATPγS molecule plays the role of reactant A, the dsDNA is the reactant B and the RecA-ATPγS-dsDNA complex represents the corresponding product, C. However, unlike in a standard bimolecular reaction, in our case, one of the reactants, the dsDNA, is an extended filament with multiple binding sites for the RecA-ATPγS molecule. Moreover, one can distinguish between two types of binding steps: 1) nucleation type where both neighboring binding sites are unoccupied and 2) growth type where at least one of the two neighboring binding sites is occupied. In what follows, we refer to such reactions as adsorption. In contrast, a simple bimolecular reaction involves two small molecules with a single binding site and its kinetics is determined by the corresponding rate constant, $k_{b}$.

Despite the apparent differences between the bimolecular adsobtion and reaction, in our case, the two become equivalent in the limit where the dsDNA filament is only three base pairs long, consisting of a single binding site for the RecA-ATPγS molecule. In this limit, the reaction kinetics is described by

$$\frac{\text{d}\left[\text{C}\right]}{\text{d}t}=k_{b}\left[\text{A}\right]\left[\text{B}\right]=k_{b}^{\prime }\left[\text{B}\right]=-\frac{\text{d}\left[\text{B}\right]}{\text{d}t}$$ (15)

where […] denotes the concentration and we have assumed that [A]≫[B], such that [A] is practically constant and $k_{b}^{\prime }=k_{b}\left[A\right]$. Since [C](t)+[B](t)=[B](0), Eq. 15 leads to an exponential growth of the products concentration, $\left[\text{C}\right]\left(t\right)=\left[\text{B}\right]\left(0\right)\left(1-e^{-k_{b}^{\prime }t}\right)$. In this case, $k_{b}^{\prime }$ and $k_{n}$ become equivalent.

Since the bimolecular adsorption and simple bimolecular reaction become equivalent in the limit described above, one expects that the exponential behavior of the latter, $\left[\text{C}\right]\left(t\right)=\left[\text{B}\right]\left(0\right)\left(1-e^{-k_{b}^{\prime }t}\right)$, can be obtained in a particular regime from the Gaussian kinetics of Eq. 4. For the adsorption, a large number of RecA-ATPγS binding sites, $L_{0}/3$, are stringed along and the decorated fraction of the dsDNA, ϕ(t), is proportional to the concentration of the products, [C](t). Therefore, there is no limit for which the Gaussian kinetics of Eq. 4 approaches the exponential kinetics of simple bimolecular reaction. However, a closer inspection of Eq. 3 reveals that in Eq. 3b should appear an additional term that accounts for the increase in the occupation of binding sites due to nucleation, $k_{n}\left(1-\phi \right)$. On one hand, such term leads to an exponential kinetics for ϕ(t) in the limit of small coverage, ϕ→0, corresponding to the short time regime before the contribution due to domain growth becomes significant. On the other hand, the $k_{n}\left(1-\phi \right)$ term is negligible unless the rate of nucleation is extremely large such that the number of nuclei is comparable to the number of sites on the dsDNA filament where nucleation can occur. This is not the case in our experiments where the number of nuclei, *N*, is typically below 10 while there are on average $\left({\bar{L}}_{0}/a\right)≅11800$ binding sites on each dsDNA molecule.

In the case of our adsorption experiments of RecA-ATPγS on dsDNA, one cannot expect the value of the average nucleation rate, $k_{n}$, to be similar to the simple bimolecular reaction rate, $k_{b}^{\prime }$. The two rates will differ for the following three reasons. First, the value of $k_{n}$ is influenced by the presence of the neighboring sections of the dsDNA on both sides of the binding site, that are absent in the case of the simple bimolecular reaction. Second, in our experiments the dsDNA molecules are tethered to the cover slip at the bottom of the sample, unlike in the simple bimolecular reaction where both reactants are free to diffuse throughout the volume of the sample. This difference between the spatial configurations of the two types of reaction leads to different collision probabilities between the reactant molecules. Finally, the value of $k_{n}$ depends on the number of binding sites on the dsDNA filament, as discussed in relation to Eq. 3, such that it should be compared to $k_{b}^{\prime }\left(L_{0}/a\right)$ rather than $k_{b}^{\prime }$ itself.

Depending on the nature of the reactants, chemical reactions can be either diffusion-limited or reaction-limited. Pugh and Cox have shown that the reaction between RecA and dsDNA is reaction-limited at saturated protein concentration (Pugh and Cox, 1987). Since our experiments are performed in this regime, the corresponding rate constants follow the Arrhenius law, that is, $k_{n}\propto \left(L_{0}/a\right)\exp\left(-\left(E_{n}/k_{B}T\right)\right)$, $k_{g}^{\prime }\propto \left(a/L_{0}\right)\exp\left(-\left(E_{gf}/k_{B}T\right)\right)$ and $rk_{g}^{\prime }\propto \left(a/L_{0}\right)\exp\left(-\left(E_{gs}/k_{B}T\right)\right)$, where $E_{n}$, $E_{gf}$ and $E_{gs}$ denote the activation energies for nucleation, fast front growth and slow front growth, respectively. These relations allow us to obtain the differences between the activation energies from the measured values of the rate constants, namely,

$$\Delta E_{coop}=E_{n}-E_{gf}=k_{B}T\ln\left(\frac{k_{g}}{\left(1+r\right)k_{n}}\frac{L_{0}^{2}}{a^{2}}\right)$$ (16)

and

$$\Delta E_{asym}=E_{gs}-E_{gf}=-k_{B}T\ln\left(r\right)$$ (17)

The value of $\Delta E_{coop}$ (see Table 1), the decrease in activation energy due to the cooperative interaction between the RecA-ATPγS molecules in the fast growing direction, is about 18 $k_{B}T$. This confirms that protein cooperativity is strong and plays a central role in determining the kinetics of the assembly of RecA-ATPγS on dsDNA (Shaner et al., 1987).

### The Asymmetry of the Domain Growth

Both the L(t) decomposition analysis allowing to determine the velocities of the slow and fast front for each individual domain and the comparison between the Monte-Carlo model and the measured average coverage kinetics, $\bar{\phi }\left(t\right)$, indicate that the domains grow asymmetrically. That is, domains grow on average about 10 times faster at their fast growing end than at the slow one. Previously, it was shown in biochemical studies that asymmetric assembly of RecA on both ssDNA and dsDNA takes place in the presence of ATP (Register and Griffith, 1985; Shaner et al., 1987; Shaner and Radding, 1987). Moreover, it was found that the fast growth is oriented in the 5′ to 3′ direction. It was suggested that the asymmetric growth of the RecA domains is due to the hydrolysis of the ATP in the RecA-ATP-DNA complex (Cox, 2007a)^.^ More recently, Galletto et al. used fluorescently labeled RecA to image the kinetics of domain growth on individual dsDNA molecules (Galletto et al., 2006). They found that while RecA domain growth is slower in the presence ATPγS than in the case when ATP is used, the domains grow asymmetrically for both. Although Galletto et al. (Galletto et al., 2006) have characterized the extent of the asymmetry in the domain growth on a mainly qualitative level, for some of their experiments with ATPγS (at particular NaCl concentrations) domain growth appears to be almost unidirectional [see Figure 4B in (Galletto et al., 2006)]. Such behavior is consistent with the behavior found in our experiments where the slow growth front of RecA-ATPγS-dsDNA is hardly advancing relative to the fast front.

Unlike in the biochemical studies, neither in our experiments nor in those of Galletto et al. (Galletto et al., 2006) can one establish the relation between the domain growth asymmetry and the direction along the DNA. However, we can use Eq. 17 to determine the difference between the energy barriers for RecA-ATPγS binding at the slow and fast ends of a domain. As was discussed above, the value of *r* obtained from the fit of the $\bar{\phi }\left(t\right)$ computed from the Monte-Carlo model to the one measured in experiments, $\bar{r}=0.10\pm 0.05$, is significantly more reliable than the value found by averaging the growth velocity ratios of individual domains, $\bar{r}=0.25\pm 0.08$. Therefore, we expect that the corresponding value of $\Delta E_{asym}$ (see Eq. 17 and Table 1), $\Delta E_{asym}=\left(2\text{.}3\pm \text{0}\text{.}5\right)k_{B}T$, represents a good estimate to the actual difference between the energy barriers for RecA-ATPγS binding at the slow and fast ends of the RecA-ATPγS-dsDNA domain.

**FIGURE 12:**
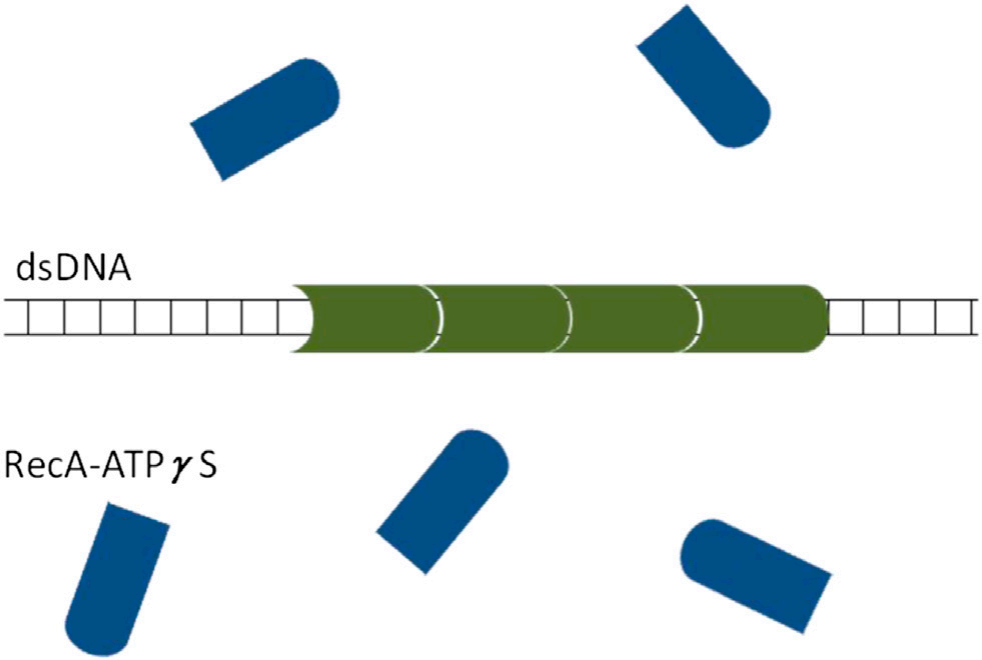
A schematic illustration of the proposed scenario leading to asymmetric RecA‐ATPγS‐dsDNA domain growth. While in solution, the RecA‐ATPγS complex (blue) has much higher binding affinity to the left end of the RecA‐ATPγS‐dsDNA domain (green) than to the right end. After binding, the RecA‐ATPγS complex changes its conformation such that its non-binding end (flat) becomes accessible to the binding of another RecA‐ATPγS complex (concave).

Regarding the mechanism of asymmetric domain growth of RecA-ATPγS on dsDNA, it may be understood assuming that the RecA-ATPγS complex undergoes a conformational change following its binding to the dsDNA (see Figure 12). Specifically, we propose that the conformation of the RecA-ATPγS complex is such that it is much more likely to bind at one end of the RecA-ATPγS-dsDNA domain than at the other. After binding however, the conformation of the RecA-ATPγS complex changes such as to allow the next RecA-ATPγS complex to bind to it. Previously, similar mechanisms were proposed to describe the treadmilling of actin filaments (Neuhaus et al., 1983). To establish the validity of the scenario depicted in Figure 12 detailed information on the structural differences between the free and the dsDNA bound RecA-ATPγS is necessary. Such analysis is beyond the scope of this study.

## Data Availability

The raw data supporting the conclusion of this article will be made available by the authors, without undue reservation.
