# Supplementary material for: Single Molecule Study of the Polymerization of RecA on dsDNA: The Dynamics of Individual Domains
Source: Front Mol Biosci. 2021 Mar 22;8:609076. doi: 10.3389/fmolb.2021.609076 (PMC8025788; doi:10.3389/fmolb.2021.609076)
Supplement: Supplementary file 1 [file table1.docx]

***Supplementay Material***

**Single Molecule Study of the Polymerization of RecA on dsDNA: the Dynamics of Individual Domains**

**Nitzan Maman^1,2^, Pramod Kumar^1,^, Amarjeet Yadav^1,3,^, and Mario Feingold^1,2, *^**

**^*^ Correspondence:** M. Feingold: mario@exchange.bgu.ac.il

**1 On the experimental monitoring the reaction between RecA and dsDNA**

Our experiments were intentionally performed in the low nucleation regime, since here we are able to use our approach to monitor the dynamics of individual RecA domain fronts. In this regime however, it is particularly difficult to obtain significant statistics. Due to the low nucleation rate, in most experiments there is no observable effect of the RecA on the bead-DNA-glass construct, that is, no change in length.

Nevertheless, we have recorded 30 different experiments in which the RecA and dsDNA reaction did occur. Out of those only 16 showed multiple step behavior. The others manifested either single step (6), continuous with no steps, probably corresponding to a large number of nuclei or ill-defined behaviors that cannot be classified and typically are due to various experimental mishaps.

Out of the 22 experiments that displayed behavior consistent with either one or few domains, only 7 (5 of which are shown in Fig. 10) where complete, in the sense that we could monitor the reaction dynamics from the start, when there is no RecA on the DNA, all the way to full coverage. However, data regarding domain statistics shown in Figs. 7 and 8 was collected from 11 experiments.

The reasons for incomplete experiments where either 1. the breaking of the tether at a time when coverage was still under way, that is, part of the DNA is still bare, 2. by the time we started monitoring the DNA length dynamics it is already larger than that of the bare DNA, suggesting that we have missed the initial stages of the RecA with dsDNA reaction, or 3. very rarely (once) a free bead would diffuse into the area of the bead-DNA-glass construct and interfere with our measurements.

**2 Extending the RecA-dsDNA complex with an approximately constant force**

During the experiments, as the RecA protein assembles on the dsDNA, the force on the trapped bead is decreasing. When a reduced force is measured, we move the stage increasing the DNA end to end length such that the initial force, 0.8 pN, is recovered. This is done either automatically, whenever the force decreases to 0.6 pN or manually via a Labview controller, whenever we note a decrease in the force. While the automatic threshold is used to prevent fast drops in the force, during most of the experiment we correct the force manually trying to maintain it as close as possible to the desired value of 0.8 pN. The observed fluctuations in the force (Fig. 3A) are thus due to the limited accuracy of the operator and the experimental error in measuring the force (the latter manifests as the rapid fluctuations in Fig. 3A).

Since most of the corrections of the DNA end-to-end distance are manual, both the time and displacement steps shown in Fig. 3B are irregular following the attempt to stay as close as possible to the constant force of 0.8 pN. We have adopted this mostly manual control approach after finding that, due to the experimental error in the force measurement, it is more accurate than the fully automatic control, maintaining the force on the DNA on average closer to the initial value. This is best illustrated in Fig. 4 where, between __ and __, presumably corresponding to the growth of the first domain, we observe several small steps in L(t), e.g. one between ~1000 to ~1200 sec and a second between ~1900 to ~2100 sec. These steps in L(t) however, are perfectly correlated with the two larger steps in y(t) (Fig. 3b) corresponding to a larger deviation from the 0.8 pN force threshold.
